# Development of the national Dutch PEWS: the challenge against heterogeneity and implementation difficulties of PEWS in the Netherlands

**DOI:** 10.1186/s12887-023-04219-3

**Published:** 2023-08-07

**Authors:** Joris Fuijkschot, Jikke Stevens, Lara Teheux, Erica de Loos, Hester Rippen, Maaike Meurs, Janke de Groot

**Affiliations:** 1 Radboud University Medical Center, Radboudumc Amalia Childrens Hospital, Nijmegen, The Netherlands; 2 Dutch Hospital Association, Utrecht, The Netherlands; 3 Dutch Foundation Child & Hospital, Utrecht, The Netherlands; 4 Netherlands Institute for Health Services Research, Utrecht, The Netherlands; 5 Knowledge Institute for Medical Specialists, Utrecht, The Netherlands

**Keywords:** Dutch PEWS, Pediatric early warning score, Risk stratification, Watcher signs, Worried sign

## Abstract

**Background:**

For the early recognition of deteriorating patients several Pediatric Early Warning Score (PEWS) systems have been developed with the assumption that early detection can prevent further deterioration. Although PEWS are widely being used in hospitals in the Netherlands, there is no national consensus on which score to use and how to embed the score into a PEWS system. This resulted in a substantial heterogeneity of PEWS systems, of which many are unvalidated or self-designed. The primary objective of this study was to develop a pragmatic consensus-based PEWS system that can be utilized in all Dutch hospitals (University Medical Centers, teaching hospitals, and general hospitals).

**Methods:**

This study is an iterative mixed-methods study. The methods from the Core Outcome Measures in Effectiveness Trials (COMET) initiative were used and consisted of two Delphi rounds, two inventories set out to all Dutch hospitals and a focus group session with parents. The study was guided by five expert meetings with different stakeholders and a final consensus meeting that resulted in a core PEWS set.

**Results:**

The first Delphi round was completed by 292 healthcare professionals, consisting of pediatric nurses and physicians. In the second Delphi round 217 healthcare professionals participated. Eventually, the core PEWS set was been developed comprising of the parameters work of breathing, respiratory rate, oxygen therapy, heart rate and capillary refill time, and AVPU (Alert, Verbal, Pain, and Unresponsive). In addition, risk stratification was added to the core set with standardized risk factors consisting of [1] worried signs from healthcare professionals and parents and [2] high-risk treatment, with the option to add applicable local defined risk factors. Lastly, the three categories of risk stratification were defined (standard, medium, and high risk) in combination with standardized actions of the professionals for each category.

**Conclusion:**

This study demonstrates a way to end a country’s struggle with PEWS heterogeneity by co-designing a national Dutch PEWS system. Currently, the power of the system is being investigated in a large multi-center study in the Netherlands.

**Supplementary Information:**

The online version contains supplementary material available at 10.1186/s12887-023-04219-3.

## Background

While pediatric hospital mortality and cardiac arrests have a low incidence, outcomes are poor and have a high impact on family and caregivers [1]. At the same time, literature shows that adverse events in pediatric care seem to occur less frequently compared to adult care, 50–60% of these events are considered preventable [2–4]. For the early recognition of deteriorating patients several Pediatric Early Warning Scores (PEWS) have been developed [5, 6]. *PEWS* generally consist of a predefined set of vital parameters, such as heart rate, respiratory rate, body temperature, blood pressure, and oxygen saturation, using age-specific cut-off points. Most PEWS also contain parameters based on behavioral items and concerns of healthcare professionals. Parameters are either added up to a numerical score that defines which response is appropriate or may directly trigger an alarm. Alarming PEWS should preclude an early intervention by healthcare professionals to prevent further deterioration. Therefore, it is essential that PEWS are part of an integrated PEWS track and trigger system in which, subsequent to the monitoring of PEWS, appropriate rules of escalation and communication are in place [7, 8].

Although PEWS are widely used in almost all Dutch hospitals there is no national consensus on which system to use [9]. Also, none of the PEWS reported in the literature has proven to be superior in the recognition of clinical deterioration [10, 11]. This may relate difficulties validating systems using quantitative endpoints such as the classically low pediatric hospital mortality This resulted in the use of a wide variety of PEWS in the Netherlands, of which many are unvalidated or self-designed. We found that 45 different systems are being used in 68 hospitals including 20 different parameters of which none is being used in all systems [12]. This large heterogeneity and use of unvalidated systems within one country are also seen in other European countries such as the United Kingdom (UK) [7]. Furthermore, while healthcare professionals in the Netherlands intuitively believe PEWS may be helpful in early recognition, they have also raised doubt due to the lack of validation and evidence of the effectiveness of PEWS and this hampers the successful implementation of PEWS in clinical practice [9, 13, 14].

The vast heterogeneity of PEWS in the Netherlands also results in a missed opportunity to validate the PEWS in different hospital settings. Due to this lack of validation, healthcare professionals in the Netherlands found it difficult to choose or develop a PEWS suitable for their local setting [9]. At the same time, a recent inventory in all hospitals in the Netherlands revealed that 98% of the hospitals would use a standardized PEWS system when available [12].

The challenge with PEWS is that clear evidence of effects upon quantitative outcome measures such as pediatric hospital mortality or unplanned admissions to Pediatric Intensive Care Units (PICU) is still lacking. Also, almost all validation studies were performed in University Medical Centers which are known for their complex population of patients with high co-morbidity and sophisticated infrastructures for the escalation of care [15]. Besides, the addition of risk stratification to PEWS has already been proven to be a powerful instrument to improve PEWS sensitivity in a University Medical Center in the Netherlands, indicating that the performance of a PEWS is related to the way it is used in clinical practice [16].

Performance studies of PEWS systems in the setting of their largest user group, general (teaching) hospitals, are largely lacking and will be very difficult to perform due to the present vast heterogeneity of systems used in countries. This will remain so unless standardization on a national level ends this heterogeneity first.

Supported by relevant Dutch scientific societies, nursing societies, and patient representatives, as well as the Dutch Healthcare Inspectorate and the Ministry of Health, we decided to start a national PEWS study.

The aimed outcome of this study was a pragmatic consensus-based system for daily practice of PEWS in Dutch hospitals.

## Methods

The main objectives of the study were to:

1. reach consensus of a Core Set-PEWS (CS-PEWS) to be used in all Dutch hospital settings, add relevant risk factors (so called watcher signs) and (possibly) risk stratification to develop a PEWS;
2. determine a set of minimal standard operating procedures for early intervention;
3. determine and limit options for adaptation of the system to the local hospital setting.

### Design and context

An iterative mixed methods study using the Core Outcome Measures Trials (COMET) initiative methodology (chosen because of its structuralized method to develop a consensus based core set) [17–19]. The study was conducted by the independent Netherlands Institute for Health Services Research in close cooperation with the Dutch Society for Pediatrics, the Dutch Society for Nursing, and Dutch Foundation Child & Hospital.

The context for this study was limited to develop a system for all children (0–18 years of age) admitted to general pediatric wards. The system is not designed for use in pediatric and neonatal intensive or high care departments and emergency departments.

Different methods and data sources were used and at times integrated. A summary of the used data sources for each objective is presented in Appendix 1.

### Study protocol

**Fig. 1:**
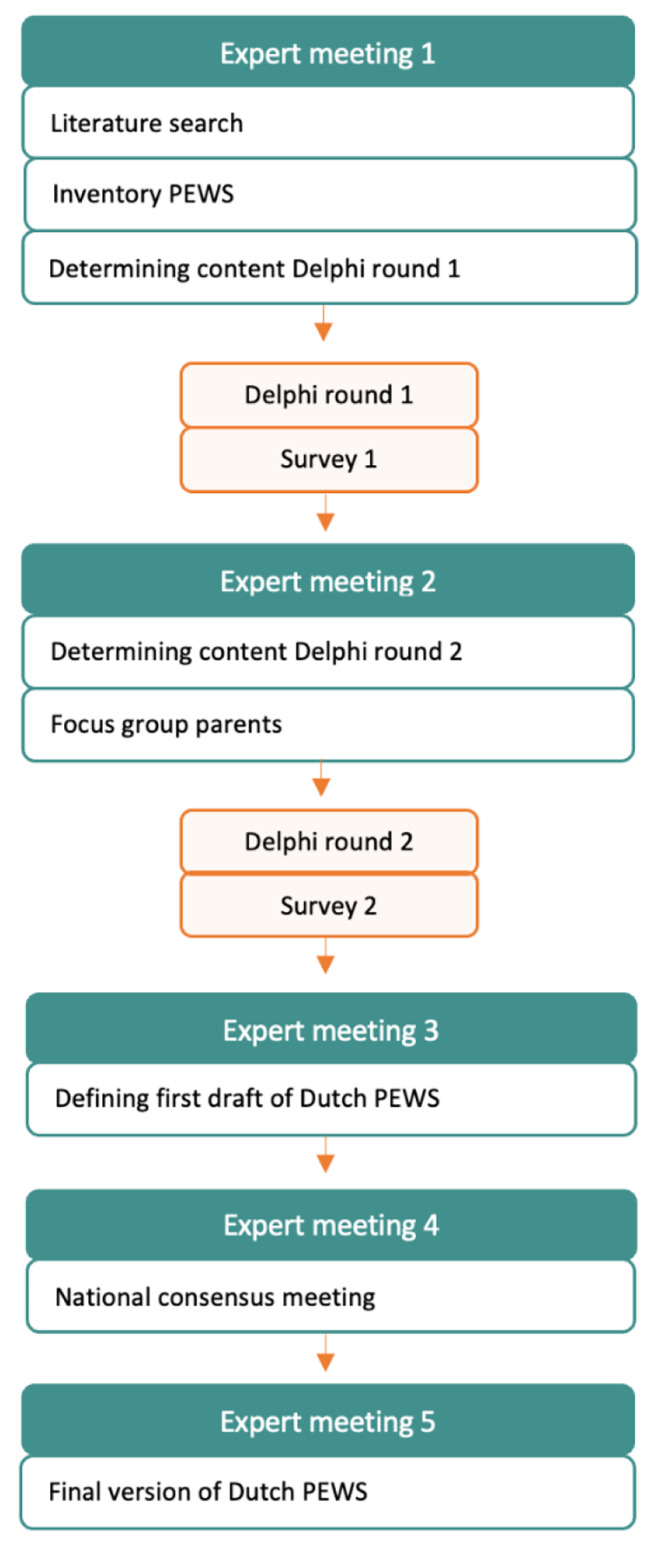
Study protocol

A summary of the iterative study protocol is summarized in Fig. 1. Appendix 2 provides a more detailed insight of the used methods.

Relevant stakeholders of PEWS involve professionals in pediatric hospital care (e.g. pediatricians, pediatric nurses, surgeons treating children), parents of children who are admitted to the hospitals, and quality improvement professionals.

During the research period, an *expert group* of stakeholders was formed with the responsibility to reach consensus in discussion and support the development of the Delphi rounds. This group consisted of experienced healthcare professionals (three pediatricians representing all different hospital settings, one pediatric surgeon, two pediatric nurses), one patient organization representative, one implementation expert, and two researchers.

All pediatricians, general surgeons treating children, and pediatric nurses in the Netherlands as well as selected patient representatives were invited through a mass mailing by their professional organizations and social media channels to participate in two *online Delphi rounds*. Reminder emails were sent to those who failed to complete any round. Parents of children were selected and invited by the Dutch Foundation Child & Hospital to participate in a *focus group session*.

The final phase of the Delphi study involved a *national consensus meeting* with representatives of all stakeholder groups including physicians, nurses, implementation professionals, parent representatives, and members of the expert group.

### Ethics

According to the Dutch Medical Research Involving Human Subject Act, this study does not require ethics approval by an independent Medical Ethics Committee. In accordance with the principles of the Declaration of Helsinki, set up by the World Medical Association (WMA), we did ask participants to give informed consent to use the data obtained during the expert meetings in an anonymous way.

## Results

### Expert meeting 1: inventory currently used PEWS parameters

The literature search resulted in five published systematic reviews on PEWS [11, 20, 21]. An inventory of PEWS parameters used in Dutch hospitals was done and has been published elsewhere [12]. Discussing both the literature and the twenty parameters currently used in Dutch hospitals, the expert group made a long list of 33 items to be included in the Delphi Survey (see Appendix 3).

### Delphi round 1 and survey

In total 212 pediatric nurses and 80 physicians completed the survey 1 (n = 292). 54 of the participants came from University Medical Centers, 85 came from teaching hospitals, and 153 came from general hospitals. Appendix 4 describes the results per item and the potential parameters to be included in the core set.

Concerning the structure of the core set, it was found that all domains (respiratory, cardiovascular and neurological; ABCD) met the present criteria for consensus inclusion (Table 1). This indicates that a core set should address all these three domains of the ABCD approach. Additional domains that were suggested by participants were ‘clinical view’, ‘cognition’, and the ‘psychological domain’.

**Table 1:** Potential ABCD domains to be included in CS-PEWS (% of answers)

	1	2	3	1 + 2 + 3	4	5	6	7	8	9	7 + 8 + 9
Respiratory (AB)	0.4	0	0	**0.4**	0	0	0	2.8	8,5	88.4	**99.7**
Cardiovascular (C)	0.3	0.3	0.3	**0.9**	0.3	0.7	0.7	4.7	15.3	71.8	**91.8**
Neurological (D)	0.7	0.3	1.3	**2.3**	0.7	3.7	2.7	12.6	20.6	51.8	**85**

9-point Likert scale: 1 ‘not important’ to 9 ‘critically important’. Items in bold met criteria for inclusion

From the initial set, the following parameters met the criteria to be included in Delphi round 2: work of breathing; respiratory rate; consciousness; heart rate; worries by physician, nurse and/or parents; pulse oxygen saturation; respiratory retractions; apnea; agitation; blood pressure; capillary refill time; skin color; body temperature, and supplemental oxygen therapy.

Participants were also asked to suggest new parameters if they felt some were missing. Additional parameters that were mentioned by participants included petechiae (mentioned twice), experienced shortness of breath (mentioned once), glucose level (mentioned once), and pupillary reflexes (mentioned once).

Furthermore, up to 79.1% of the participants indicated that “worried signs” should be considered as a separate acting alarm sign and by itself be able to directly trigger a response. In regards to the optimal number of items in a PEWS, the participants agreed that the final core set should consist of five to seven preferable non-invasive parameters.

### Expert meeting 2: prioritizing items for the next round

Based on the survey with only limited suggestions for additional parameters, the expert group concluded that the long list was complete and results from Delphi round 1 were considered valid. All of the included parameters therefore were eligible for Delphi round 2. The importance to address all three of the ABCD domains in the core set was confirmed. Cognition and psychological domain were considered to be part of the neurological domain and subjective signs were already taken into account with the worried signs. Therefore, no complementary domains were added to the structure of the core set.

Relating to worried signs, it was decided that the worries by physicians, nurses or parents should not be prioritized in the second Delphi but were considered eligible to act as a separate risk factor in the system. Its exact position and role were determined later in the study.

### Delphi round 2 and survey

#### Part 1: prioritizing shortlist

In the second survey participants (n = 217 - a full subset of survey 1) were asked to further prioritize parameters to be able to create a core set of eight items (see Appendix 5 for full results). This resulted in the following high to low order meeting inclusion criteria: heart rate (95.5%), work of breathing (92%), pulse oxygen saturation (89.9%), respiratory rate (89%), consciousness (88%), supplemental oxygen therapy (78.3%), capillary refill time (74.6%), and agitation (71%).

#### Part 2: prioritizing within three domains

Participants were also asked to rank the remaining parameters included in survey 1 within the respiratory, cardiovascular and neurological domains.

In the respiratory domain five parameters were being prioritized. Work of breathing was considered the most important parameter, followed by respiratory rate, apnea, oxygen saturation, and supplemental oxygen therapy (see Table 2).

**Table 2:** Ranking of parameters of respiratory domain (%)

	1	2	3	4	5
Work of breathing	8.8	12.6	14.9	20.9	**42.8**
Respiratory rate	11.2	15.8	27.9	27.0	**18.1**
Pulse oxygen saturation	5.1	18.1	38.1	26.0	**12.6**
Apnea	35.5	22.9	8.9	13.6	**19.2**
0_2_therapy	39.5	30.7	10.2	12.6	**7.0**

Within the cardiovascular four parameters were being prioritized. Heart rate was considered most important, followed by capillary refill time, blood pressure, and skin color (see Table 3).

**Table 3:** Ranking of parameters of the cardiovascular domain (%)

	1	2	3	4
Heart rate	14.4	10.7	14.0	**60.9**
Capillary refill time	10.2	33.0	40.5	**16.3**
Blood pressure	37.2	26.0	24.2	**12.6**
Color of skin	38.1	30.2	21.4	**10.2**

Within the neurologic domain (consisting of agitation and consciousness), consciousness was considered the most important parameter. The temperature was not taken into account as this is not a parameter relating to the ABCD but to the E domain of the ABCDE approach.

#### Survey on scoring, implementation and organization of working with a PEWS

Next to prioritizing items, the survey also included questions on risk factors to be taken into account and how to use PEWS in different hospital settings. Respondents prioritized the following items as important risk factors: medical history of the patient, high-risk medication, abnormal airway, and high-risk interventions (see Appendix 6).

When asked about which patients, how often, and when to measure PEWS (Appendix 7), most respondents answered PEWS should be applied to all hospitalized children (52.4%), with a large group (31.6%) thinking this should depend on factors such as diagnosis, disease severity, high-risk patient, threatened vital signs, gut feeling, clinical point of view, surgery, worried parents, admission in the maternity departments. More specifically there were questions about whether PEWS are relevant for children admitted for psychosocial reasons or observations.

Most respondents agreed PEWS should ideally be assessed three times per 24 h, at the beginning of each nursing shift, while again a large group (30.6%) answered this should depend on factors such as diagnosis, indication for admission, disease severity, time after admission, clinical point of view, previous PEWS scores and whether a child is sleeping. With regard to sleep, only 2.4% thought children should always be awakened for the assessment of PEWS, while over 80% thought this should depend on the circumstances.

#### Focus group session

A total of five parents (representing five children) participated in the focus group session. They expressed the wish for a lean system using mostly observations while limiting the number of invasive measurements to lower the impact of the repeated PEWS measurements on their children. Wherever possible they would like to be part of the PEWS measurement to help their children to feel as comfortable as possible and maybe improve diagnostic accuracy. The participants of the focus group session stated that the worried sign given by parents is of utmost importance to be taken into account when developing a new system. They shared their experiences in recognizing a deviant clinical course during the admissions of their children. Their ‘gut-feeling’ was often felt as a strong predictor for clinical deterioration and therefore should play part in a PEWS.

### Expert meeting 3: first draft of Dutch PEWS

**Fig. 2:**
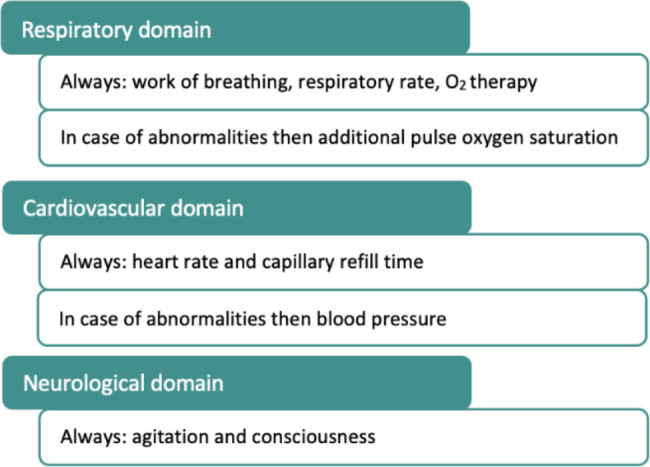
First draft of the Dutch PEWS

This meeting focused on constructing a first draft of PEWS. The primary goal was the concept of a lean, minimal invasive, child-friendly and nurses workload reducing system. Firstly, based on the second Delphi round and the focus group session, the Dutch PEWS core set was formulated. It was decided to use eight included parameters but also to differentiate between ‘early’ and ‘late’ predictors of clinical deterioration. The differentiation was based on physiological principles of bodily responses to clinical deterioration. For the respiratory domain, it was decided that the work of breathing and respiratory rate are the most important ‘early’ predictors. Pulse oxygen saturation usually only decreases after attempts to compensate for respiratory failure have failed. In the absence of increased respiratory rate and/or work of breathing, it is unlikely that pulse oxygen saturation is decreased. Therefore, pulse oxygen saturation is considered a late predictor. For the cardiovascular domain, the early predictors were considered to be the heart rate and capillary refill time since these parameters are already deviant in early, compensated shock. In critically ill children, blood pressure often is compensated for a long time hence this was considered to be a late predictor of clinical deterioration [22]. For the neurological domain, a differentiation between early and late parameters could not be made so both parameters were included in this draft of the core set. This resulted in the following first draft of the PEWS core set, summarized in Fig. 2.

Secondly, the expert group discussed the position of risk factors (watcher signs) that may or may not directly trigger an alarm and the principle of risk stratification. Risk factors often are present early during admission and may improve PEWS’ sensitivity to detect clinical deterioration if used to stratify patients into risk categories. Risk stratification enables professionals to follow up on watcher patients more closely with more checks and measurements whilst non-watcher patients receive standard care. This may lead up to a more refined system, refined effort of human resources, and patient friendliness. Previous experience with risk factors and risk stratification in the Netherlands found a significant increase in PEWS sensitivity as well as positive effects on communication and situational awareness [13, 16]. Based upon Delphi rounds data, literature and national experiences, it was decided that risk factors and risk stratification are crucial components of the Dutch PEWS system. To determine its exact position and usage in the system the subsequent consensus meeting was needed to further receive input from the working field.

### Expert meeting 4: national consensus meeting

During this consensus meeting, the first draft of the Dutch PEWS and data from the survey was presented and discussed with a large multidisciplinary group of professionals from twenty different hospitals (including four University Medical Centers, seven teaching hospitals, and nine general hospitals) and patient representatives. Using the methodology of ‘world café’ [23] the group was asked in several rounds to comment and (dis)agree upon the first draft, advice on risk factors and risk stratification, formulate which children should be assessed, how often and when, and what actions should be part of the rapid response to an alarming PEWS score.

Outcomes resulted in the following recommendations for the expert group:

- The first draft of the core set was fully supported by the participants. For the neurological domain, it was advised to use the AVPU (Alert, Verbal, Pain, and Unresponsive) score for consciousness. Agitation was not recognized as a crucial PEWS component since this usually coincides with alternations in consciousness. It was advised to use data from Parshuram’s Bedside PEWS to determine cut-off points for different PEWS parameters and age-groups since this is a rather sufficiently validated set.
- Risk factors (watcher signs) should consist of standardized factors (such as worried signs and high-risk treatment) and part of the core set but also supplemented by locally defined non-core set risk factors to improve the adaptability of the system to the local hospital.
- Risk stratification should be applied to identify watcher patients. Three risk categories (high, medium and standard) were defined and recommendations for minimal actions of the professionals were defined for each risk category.
- PEWS measurements apply to every patient admitted to the general pediatric department (including the day care unit) as part of standard care. Exclusions can be made if deemed necessary by the healthcare professionals.
- The standard care will consist of three PEWS measurements per 24 h, including the assessment of ‘watcher signs’ once per day. The timing of the PEWS measurements can be integrated with the nurses rounds and ideally patients are not wakened during the night.
- Parents should be informed about PEWS and asked about possible worries they have.
- Subsequent validation of Dutch PEWS was highly recommended.

### Expert meeting 5: final version of Dutch PEWS

The final expert meeting focused on the last issues to complete the Dutch PEWS. For the core set part of the system, based upon input from the consensus meeting, it was decided to use the data from the Parshuram bedside PEWS (normal values for different age groups) as this was considered to be the most validated set available at that time. Numeric scoring thresholds for the sum of the respiratory and cardiovascular domains were defined and divided into three scoring groups (0–3/4–6/≥7) with an alarming score threshold at seven points. The threshold is based on previous experiences with other PEWS systems in the Netherlands that also used these parameters [16].

The use of the AVPU score for the neurological domain was approved. It was decided to position this score as a risk factor. This was decided because the sensitivity of PEWS to timely detect patients with abnormal consciousness is notoriously low [16]. With the change to risk factor, abnormal consciousness will automatically move a patient from the standard to high-risk assessment independent from other parameters.

**Fig. 3:**
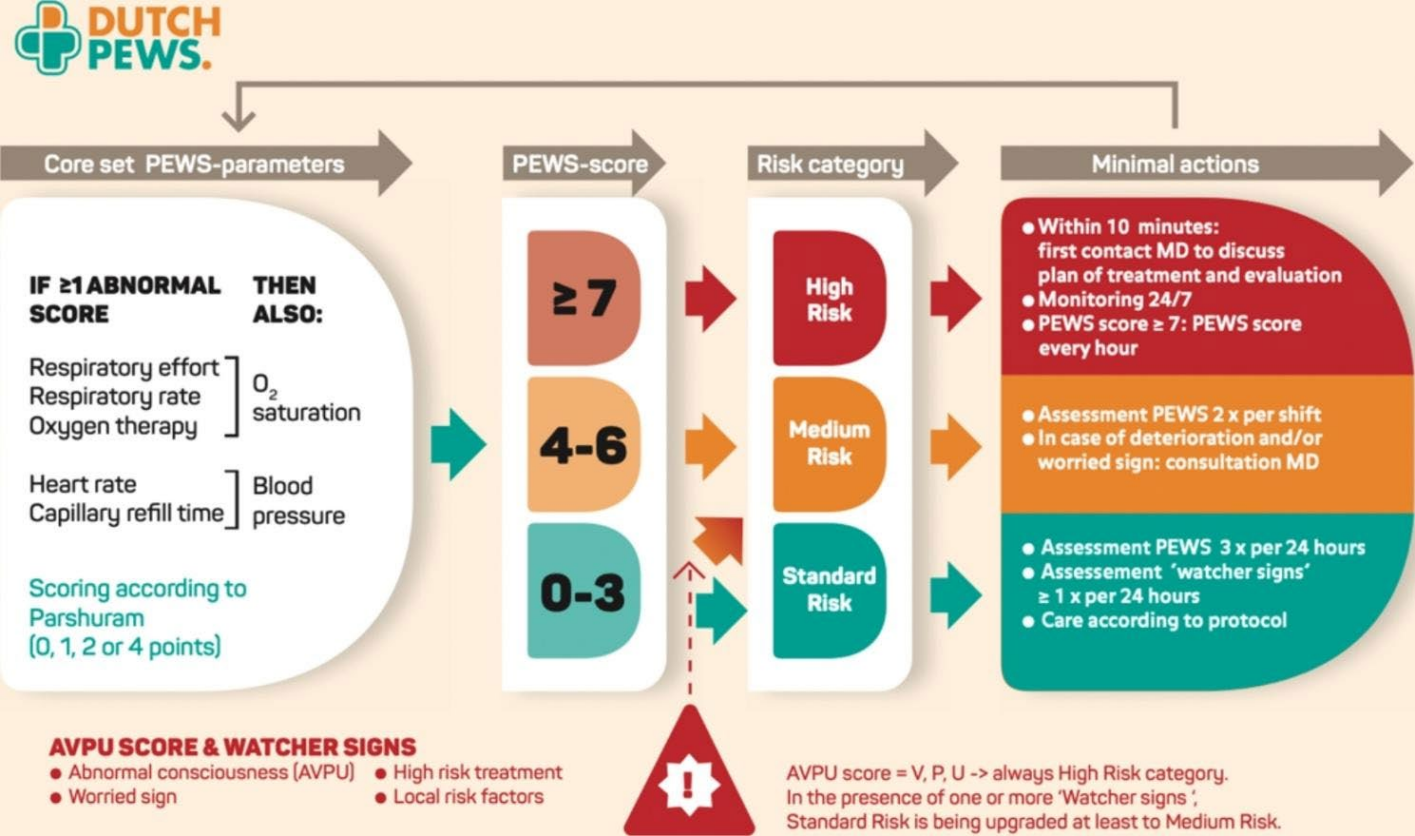
Dutch PEWS and actions

Further attention was paid to the role of risk factors and the application of risk stratification. In the final version of the Dutch PEWS, the numeric PEWS score and the existence of watcher signs (including AVPU and worried signs) adds up to a risk score, ranging from standard, medium to high, with specific recommendations for action (see Fig. 3).

For further approval of this newly designed and unique system, the results were presented and discussed at the annual meeting of the Dutch Pediatric Society (2019) and the Dutch Society for Pediatric Nurses (2020) and presented to the Dutch Healthcare Inspectorate (2020). In all these meetings relevant stakeholders approved the system for usage with a strong recommendation for subsequent validation of the system in Dutch hospitals.

For implementation and promotion purposes an infographic was made and a website with information about the Dutch PEWS (www.dutchpews.com) to enable the usage of the new system in the Netherlands.

## Discussion

In the context of the heterogeneity of currently used PEWS systems in different countries over the world, we demonstrate a way to end this struggle by co-designing a consensus-based PEWS suitable for all hospital settings. To the best of our knowledge, this study is unique using Delphi methodology with a large group of professionals and patients/parents to construct a national PEWS system and not reported by other countries. Unique in relation to other national systemsis the participation of a large number of pediatric healthcare professionals using Delphi methods and paying attention to local hospital context differences that may influence PEWS performance and implementation. Further, the consensus-based PEWS is expected to fit well within the current practice in Dutch pediatric care. The approach of this study can serve as an example to solve problems with heterogeneity in PEWS systems in other countries.

In the design of the system a lot of attention has been given to feasibility in daily practice (e.g. the time consuming and child unfriendly parameters blood pressure and pulse oxygen saturation are only to be measured when other less challenging PEWS parameters are abnormal). This increases the expectations of the Dutch PEWS to meet its original objectives and at the same time be child friendly and reduce nurses’ workload. The time-consuming use of technical medical equipment in otherwise stable patients is eliminated. It is expected that this helps to improve protocol adherence, which is a notorious problem in PEWS system implementation [14].

To facilitate implementation and protocol adherence, preparations have been made by the study group to incorporate the Dutch PEWS system in the electronic health record systems of the two largest providing companies in the Netherlands; Epic and ChipSoft. Working together with representatives of these providers and the national network of chief medical information officers in the Netherlands in 2020, led to the direct availability of this national system for approximately 85% of all hospitals. In its design, much attention has been paid to reducing registration load and improving the usability of the data in a user-friendly interface.

To the best of our knowledge, the Dutch PEWS system is the first nationwide PEWS that incorporates risk stratification and explicitly shifts focus to those patients of whom a deviant clinical course can be expected (so-called ‘watcher patients’). Risk stratification can help identify these watcher patients and enables professionals to pro-actively follow up on their clinical course more closely and respond to deterioration more quickly.

### Limitations

The largest limitation of this study is that by designing a new system it is unclear what the power of the system is in terms of sensitivity and diagnostic accuracy. Parameters from Delphi round 1 were scored upon clinical relevance in participants’ opinion/experience and this may not reflect importance in terms of validity in its assessment [24]. Although elements of the existing and validated Bedside PEWS were used and the basis of the system resembles a well-studied system that has been used for many years in a University Medical Center in the Netherlands (Radboud University Medical Center), it is of utmost importance that the system is validated in the different hospital settings for which it is designed.

Furthermore, there is a risk of selection bias amongst participants of the Delphi rounds by including mainly believers or non-believers of a PEWS system. However, due to the rather large amount of respondents, selection bias probably had limited influence on the results of the Delphi rounds.

### Future research

Currently, a large multi-center study is being performed in the Netherlands testing the system in twelve hospitals and one ambulance service. These twelve hospitals include three University Medical Centers (comprising one national pediatric oncology center), five teaching hospitals, and four general hospitals. The main objectives are to validate the system using different endpoints customized for hospital setting and to determine its effectiveness in improving patient safety. The results and findings of this study are expected in 2024. Experiences from hospitals participating in this study need to be actively shared with all hospitals in the Netherlands to provide the opportunity to learn and improve together. More information regarding this study is available at the website https://dutchpews.com.

## Conclusion

By co-designing a nationwide and consensus-based PEWS suitable for all hospital settings, this study contributes to the quality and patient safety of pediatric care. The study protocol can serve as a blueprint and support other countries to solve problems with heterogeneity in PEWS systems and enable subsequent studies focusing on the validity and effectiveness of the system nationwide. Currently, the power of the Dutch PEWS is being investigated by this research group in a large multi-center study in the Netherlands.

### Electronic supplementary material

Below is the link to the electronic supplementary material.

Supplementary Material 1

## Data Availability

The datasets used and/or analysed during the current study are available from the corresponding author on reasonable request.

## List of abbreviations

ABCDE: Airway, Breathing, Circulation, Disability, and Exposure
AVPU: Alert, Verbal, Pain, and Unresponsive
COMET: Core Outcome Measures in Effectiveness Trials
Kind&Ziekenhuis: Child and Hospital Foundation
Nivel: Netherlands Institute for Health Services Research
NVK: Dutch society for pediatrics
PEWS: Pediatric Early Warning System
PICU: Pediatric Intensive Care Unit
PMET: Pediatric Medical Emergency Team
V&VN: Dutch Society for Nursing
VMS: National safety program
