# Supplementary material for: Development of the national Dutch PEWS: the challenge against heterogeneity and implementation difficulties of PEWS in the Netherlands
Source: BMC Pediatr. 2023 Aug 7;23:387. doi: 10.1186/s12887-023-04219-3 (PMC10405440; doi:10.1186/s12887-023-04219-3)
Supplement: Supplementary file 1 — Supplementary Material 1 [file 12887_2023_4219_MOESM1_ESM.docx]

# Appendices

| **Objectives** | **Data sources** |
| --- | --- |
| Consensus of a CS-PEWS | Literature review Inventory PEWS in Dutch hospitals Two digital Delphi rounds One focus group with parents Five expert meetings One national consensus meeting |
| Consensus additional risk factors and risk stratification | Literature review Inventory PEWS in Dutch hospitals One focus group with parents Survey questions Five expert meetings One national consensus meeting |
| Options for local adaptation | Survey questions Five expert meetings One national consensus meeting |
| Definition of standard operating procedures | Five expert meetings One national consensus meeting |

**Appendix 1.** Objectives and data sources

**Appendix 2.** Detailed description of the used methods

Inventory currently used PEWS A comprehensive literature search was performed to identify currently used PEWS parameters, clinical outcomes, patient-reported outcomes, and instruments that are used in hospitals to measure the PEWS criteria for the management and follow-up of deteriorated children. The search strategy was applied to the Cochrane Library, PubMed, and Embase. The search included systematic reviews, published in English or Dutch between January 2010 and February 2018. Additionally, an inventory regarding used PEWS in Dutch hospitals had been performed in preparation for this study (12). The parameters obtained from the literature search and the Dutch inventory were used to design the first Delphi round.

Delphi CS-PEWS We conducted a two-round Delphi study guided by the recommendations of COMET, using a web-based survey system from the Nivel institute. Each round was open for 21 days and a reminder was sent after ten days. A short questionnaire was included in the first round for demographics, profession, work environment, and work experience in pediatric care. The list of parameters was listed alphabetically. Participants were asked to rate the importance of a list of these potential parameters to be included in the CS-PEWS. Definitions of parameters were not given separately but were based upon professional training standards such as Advanced Pediatric Life Support (APLS). Participants were asked to score each of the symptoms and outcomes listed using a 9-point Likert scale (17,24). In this Delphi exercise the scale was presented with 1 to 3 labeled as ‘not important’, and 7 to 9 labeled as ‘very important’. Parameters rated by more than 50% of the participants as ‘very important’ (7-9) and by less than 15% of the
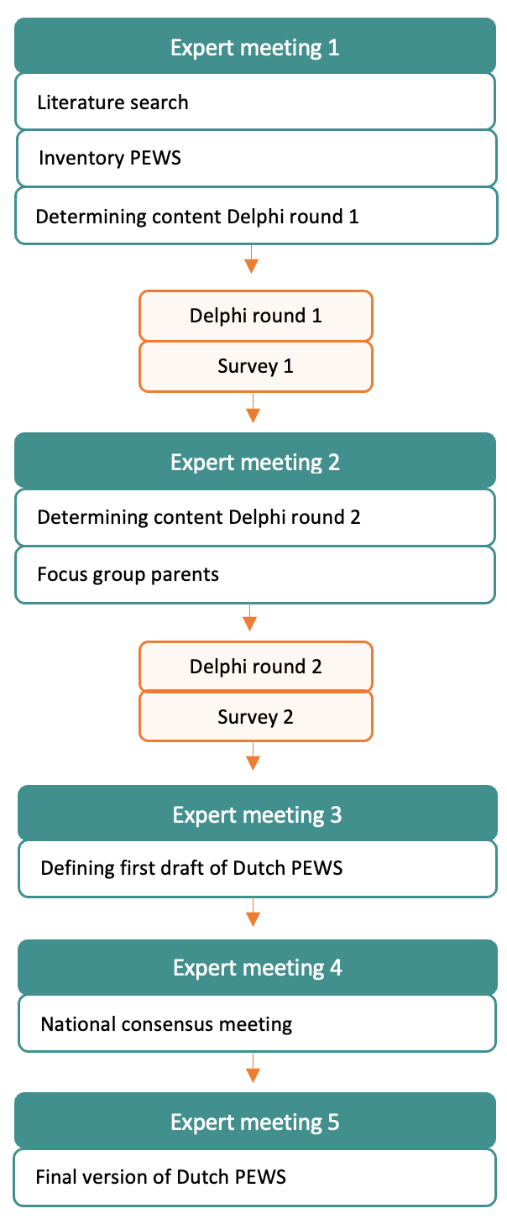
participants as ‘not important’ (1-3) moved on to the second Delphi round. Additional criteria/outcomes listed by participants were reviewed and coded by two members of the study team (EdL and JdG) to ensure they represented new outcomes. These were discussed in the expert meeting.

**Figure 1.** Study Protocol

Furthermore, the structure of the PEWS needed to be defined. Most PEWS systems structure parameters according to the Airway, Breathing, Circulation, Disability, and Exposure (ABCDE) approach. The ABCDE approach is a universal, priority-based approach for the initial assessment and treatment of critically ill patients. PEWS parameters can be classified accordingly into a respiratory (AB), cardiovascular (C) and neurological (D) domain. To establish the importance of all sub-domains to be represented in the core set, the participants were asked to score the relevance of these separate domains.

During the second Delphi round participants were invited to further prioritize the remaining parameters individually, but also within their different domains (respiratory-, cardiovascular- and neurological parameters). In this second Delphi round consensus was defined as >70% 7-9 scores and <15% 1-3 scores. The participants in this round were a subset of the participants of Delphi round 1. Further permission of the participants to be invited to a large national consensus meeting was asked. Initially, a third Delphi round was planned for, but not needed as consensus was easily reached after two rounds.

Focus group interview parents The parents representative group, Dutch Foundation Child & Hospital, highly recommended a focus group session over-involvement of parents in the digital Delphi rounds. Parents of children with multiple admissions to the hospital over the last few years were invited through social media and a newsletter from the Dutch Foundation Child & Hospital. The following topics were discussed during the focus group:

- background and short introduction PEWS;

- type and frequency of daily assessment;

- possible role of parents in PEWS assessment;

- perceptions and personal experience PEWS assessment;

- recommendations about PEWS system.

One researcher moderated the session, while another took notes and asked additional questions to ensure full discussion of the prepared topic list. The meeting was recorded and a detailed report was made and sent back to the participants for comprehensiveness and correctness. The report was then fragmented and coded by two researchers. Outcomes were discussed during expert meeting 3.

Survey for daily use, implementation and validation of PEWS Together with the ranking for parameters to be included in the core set, a survey was sent out in two parts. The first part included questions about ideal daily use for PEWS. Questions were related to the patient population, frequency of PEWS assessment, when to assess, how many parameters to include in a core set, contextual factors to include in relation to risk stratification etc. The second part was sent out with Delphi round 2 and included questions related to facilitators and barriers for the implementation of PEWS, the role of ‘worried sign’, and possible outcomes measures for future validation of PEWS especially aimed at the setting of a general hospital.

National consensus meeting During the national consensus meeting the results from the two Delphi rounds and surveys were presented in detail. During an eight-hour meeting, participants were invited through interactive short work sessions to reach consensus on the CS-PEWS, the scoring system, relevant contextual factors, risk stratification, and follow-up action based on the risk stratification. This resulted in a concept core set and system of PEWS for all Dutch hospitals. A comprehensive report was written and sent back to all participants for comprehensiveness and correctness.

**Appendix 3.** Parameters from literature, Dutch hospitals and inclusion in survey 1

| **Parameters from literature** | **Inventory Dutch Hospitals** | **Final list for survey (long list)** |
| --- | --- | --- |
| Respiratory rate | Respiratory rate | Respiratory rate |
| Work of breathing | Work of breathing | Work of breathing |
| Respiratory retractions | Respiratory retractions | Respiratory retractions |
| O_2_ therapy | O_2_ therapy | O_2_ therapy |
| O_2_ saturation | O_2_ saturation | O_2_ saturation |
| Apnea |  | Apnea |
| Nebulization | Nebulization | Nebulization |
| Respiratory arrest |  |  |
| Stridor |  | Stridor |
| Wheezing |  | Wheezing |
| Heart rate | Heart rate | Heart rate |
| Blood pressure | Blood pressure | Blood pressure |
| Blood pressure (systolic) |  | Blood pressure (systolic) |
| Capillary refill time | Capillary refill time | Capillary refill time |
| Consciousness | Consciousness | Consciousness |
| Agitation |  | Agitation |
| Seizures | Seizures | Seizures |
| Diarrhea | Diarrhea | Diarrhea |
| Urinary production | Urinary production | Urinary production |
| Vomiting |  | Vomiting |
| Behavior | Behavior |  |
| Skin color | Skin color | Skin color |
| Body temperature | Body temperature | Body temperature |
| Worries physician | Worries physician | Worries physician |
| Worries family members |  |  |
| Worries parents | Worries parents | Worries parents |
| Worries nurse | Worries nurse | Worries nurse |
| Pain score | Pain score | Pain score |
| Continued post-operative vomiting | Continued post-operative vomiting |  |
| Shock |  | Shock |
| Drowsiness |  |  |
| Central cyanosis |  | Central cyanosis |
| Hyperkaliemia |  | Hyperkalemia |
| Suspected meningococcal disease |  | Suspected meningococcal disease |
| Diabetic ketoacidosis |  | Diabetic ketoacidosis |
| pH |  | pH |
| Lactate |  | Lactate |
| Pediatric Index of Mortality (PIM 2) |  | Pediatric Index of Mortality (PIM 2) |

**Appendix 4.** Potential parameters to be included in the CS-PEWS (%)

| n=292 | **1** | **2** | **3** | **1+2+3** | **4** | **5** | **6** | **7** | **8** | **9** | **7+8+9** |
| --- | --- | --- | --- | --- | --- | --- | --- | --- | --- | --- | --- |
| **Work of breathing** | 1.9 | 0.0 | 0.4 | **2.3** | 0.4 | 1.5 | 2.2 | 3.7 | 17.2 | 72.7 | **93.6** |
| **Retractions** | 7.9 | 3.0 | 3.0 | **13.9** | 4.5 | 9.0 | 5.2 | 17.2 | 12.7 | 37.5 | **67.4** |
| **Respiratory rate** | 0.7 | 0.7 | 0.0 | **1.4** | 0.7 | 1.5 | 2.6 | 8.6 | 20.6 | 64.4 | **93.6** |
| **Apnea** | 7.5 | 3.0 | 4.5 | **15** | 5.6 | 7.9 | 7.9 | 13.9 | 16.1 | 33.7 | **63.7** |
| Stridor | 4.9 | 3.7 | 7.9 | 16.5 | 6.7 | 12.4 | 13.9 | 20.2 | 14.2 | 16.1 | **50.5** |
| Wheezing | 7.9 | 3.7 | 7.1 | 18.7 | 9.4 | 15.4 | 14.6 | 20.6 | 11.2 | 10.1 | 41.9 |
| **Agitation** | 2.6 | 2.6 | 0.4 | **5.6** | 1.1 | 8.6 | 6.0 | 14.2 | 25.8 | 38.6 | **78.6** |
| **Consciousness** | 1.1 | 0 | 0.4 | **1.5** | 1.5 | 2.6 | 2.2 | 11.2 | 21.0 | 59.9 | **92.1** |
| **Blood pressure** | 6.4 | 3.7 | 4.9 | **15** | 7.1 | 13.1 | 12.7 | 21.3 | 10.9 | 19.9 | **52.1** |
| Blood pressure  (systolic) | 11.2 | 4.1 | 5.2 | 20.5 | 7.1 | 18.0 | 12.4 | 18 | 12 | 12 | 42 |
| Vomiting | 21.0 | 12.7 | 15.7 | 49.4 | 9.4 | 16.5 | 10.5 | 10.9 | 2.2 | 1.1 | 14.2 |
| **Capillary refill time** | 1.9 | 1.9 | 2.6 | **6.4** | 3.4 | 6.4 | 5.2 | 16.1 | 23.6 | 39.0 | **78.7** |
| Convulsion | 15.0 | 8.6 | 6.4 | 30 | 6.7 | 15.0 | 8.2 | 12.4 | 12.4 | 15.4 | 40.2 |
| Diarrhea | 26.6 | 15.4 | 9.7 | 51.7 | 9.0 | 18 | 7.5 | 10.1 | 2.2 | 1.5 | 13.8 |
| **Urinary production** | 12.0 | 6.4 | 4.9 | 23.3 | 6.7 | 15.7 | 12 | 18 | 13.9 | 10.5 | 42.4 |
| **Heart rate** | 0.7 | 0 | 0 | **0.7** | 0.4 | 0.7 | 1.5 | 4.1 | 22.8 | 69.7 | **96.6** |
| **Color of skin** | 4.1 | 2.2 | 3.0 | **9.3** | 4.1 | 7.1 | 9.4 | 16.9 | 23.6 | 29.6 | **70.1** |
| **Temperature** | 2.2 | 3.4 | 2.2 | **7.8** | 4.5 | 7.9 | 9.4 | 22.1 | 19.1 | 29.2 | **70.4** |
| **Worries physician** | 7.9 | 2.2 | 2.2 | **12.3** | 3.7 | 9.0 | 9.4 | 15.7 | 20.6 | 29.2 | **65.5** |
| **Worries parents** | 2.2 | 1.5 | 2.6 | **6.3** | 1.5 | 6.0 | 11.2 | 16.5 | 24.7 | 33.7 | **74.9** |
| **Worries nurse** | 1.9 | 0 | 1.5 | **3.4** | 1.1 | 4.9 | 6.4 | 14.6 | 27.0 | 42.7 | **84.3** |
| Pain score | 11.2 | 7.9 | 7.1 | 26.2 | 7.1 | 16.1 | 13.1 | 18.7 | 9.7 | 9.0 | 37.4 |
| **O_2_ saturation** | 0.7 | 0.7 | 0.7 | **2.1** | 0 | 2.6 | 2.6 | 9.4 | 27.0 | 56.2 | **92.6** |
| Shock | 16.1 | 5.6 | 5.2 | 26.9 | 4.9 | 12.0 | 6.0 | 10.5 | 9.4 | 30.3 | **50.2** |
| Nebulization | 24.3 | 10.5 | 10.1 | 44.9 | 9 | 18 | 9.4 | 11.6 | 6.7 | 0.4 | 18.7 |
| **O_2_ therapy** | 4.1 | 1.9 | 3.0 | **9** | 2.2 | 6.7 | 7.5 | 19.5 | 21 | 34.1 | **74.6** |
| Central cyanosis | 10.1 | 3.7 | 5.6 | 19.4 | 6.0 | 8.6 | 9.0 | 12.4 | 19.5 | 25.1 | 57 |
| Hyperkalemia | 24.0 | 10.9 | 7.1 | 42 | 9.7 | 15.7 | 9.7 | 10.1 | 9.0 | 3.7 | 22.8 |
| Suspected meningococcal disease | 25.1 | 9.4 | 7.5 | 42 | 7.9 | 15.4 | 6.0 | 9.4 | 7.9 | 11.6 | 28.9 |
| Diabetic ketoacidosis | 25.5 | 8.6 | 8.2 | 42.3 | 7.9 | 14.6 | 7.1 | 10.5 | 9.7 | 7.9 | 28.1 |
| pH (blood gas) | 25.8 | 9.0 | 8.6 | 43.4 | 6.4 | 11.6 | 8.6 | 15.0 | 9.0 | 6.0 | 30 |
| Lactate | 27.0 | 9.7 | 8.6 | 45.3 | 6.4 | 12.4 | 8.6 | 15.4 | 9.4 | 2.6 | 27.4 |
| PIM2 score | 29.6 | 9.7 | 6.7 | 46 | 7.9 | 15.4 | 7.5 | 13.1 | 6.0 | 4.1 | 23.2 |

9-point Likert scale: 1 ‘not important’ to 9 ‘critically important’. Items in bold met criteria for inclusion.

**Appendix 5.** Prioritizing outcomes Delphi round 2 (%)

| n=217 | **1** | **2** | **3** | **1+2+3** | **4** | **5** | **6** | **7** | **8** | **9** | **7+8+9** |
| --- | --- | --- | --- | --- | --- | --- | --- | --- | --- | --- | --- |
| **Work of breathing** | 0.5 | 1.4 | 0.9 | **2.8** | 0 | 2.3 | 2.8 | 4.6 | 12.9 | 74.7 | **92** |
| **Respiratory rate** | 0.5 | 0 | 1.4 | **1.9** | 0 | 2.8 | 6.5 | 10.6 | 18.0 | 60.4 | **89** |
| Apnea | 3.7 | 3.2 | 6.5 | **13.4** | 3.7 | 12.4 | 6.0 | 15.2 | 15.7 | 33.6 | 64.5 |
| **Agitation** | 0.9 | 0.9 | 3.7 | **5.5** | 4.1 | 10.1 | 9.2 | 24.0 | 23.0 | 24.0 | **71** |
| **Consciousness** | 0.5 | 0.5 | 0.5 | **1.5** | 1.4 | 6.0 | 3.2 | 14.3 | 23.0 | 50.7 | **88** |
| Blood pressure | 2.3 | 6.5 | 8.8 | 17.6 | 7.4 | 14.7 | 12.4 | 15.7 | 13.4 | 18.9 | 48 |
| **Capillary refill time** | 0.5 | 1.4 | 1.4 | **3.3** | 5.1 | 7.4 | 9.7 | 20.7 | 22.1 | 31.8 | **74.6** |
| **Heart rate** | 0.5 | 0.0 | 0.0 | **0.5** | 0.9 | 0.9 | 2.3 | 11.1 | 20.3 | 64.1 | **95.5** |
| Color of skin | 2.3 | 0.9 | 4.6 | **7.8** | 6.5 | 14.7 | 12.0 | 20.3 | 18.4 | 20.3 | 59 |
| Temperature | 0.9 | 2.3 | 2.3 | **5.5** | 8.8 | 16.6 | 10.1 | 20.7 | 16.1 | 22.1 | 58.9 |
| **O_2_ saturation** | 0.0 | 0.5 | 1.4 | **1.9** | 0.5 | 3.7 | 4.1 | 8.8 | 18.9 | 62.2 | **89.9** |
| **O_2_ therapy** | 0.9 | 1.8 | 2.8 | **5.5** | 1.4 | 7.8 | 6.9 | 19.8 | 22.1 | 36.4 | **78.3** |

9-point Likert scale: 1 ‘not important’ to 9 ‘critically important’. Items in bold met criteria for inclusion.

**Appendix 6.** The contextual factors should be taken into account when interpreting PEWS scores (%)

| n=217 | **1** | **2** | **3** | **1+2+3** | **4** | **5** | **6** | **7** | **8** | **9** | **7+8+9** |
| --- | --- | --- | --- | --- | --- | --- | --- | --- | --- | --- | --- |
| **Diagnosis** | 5.9 | 1.6 | 2.4 | **9.9** | 2.0 | 5.9 | 6.3 | 17.6 | 21.6 | 36.9 | **76.1** |
| **High-risk medication** | 5.1 | 1.6 | 3.9 | **10.6** | 2.7 | 8.6 | 9 | 21.6 | 25.5 | 22.0 | **69.1** |
| **Laboratory values** | 7.1 | 2.4 | 4.7 | **14.2** | 3.9 | 11.4 | 19.6 | 23.9 | 16.1 | 11.0 | 51 |
| **Abnormal airway** | 4.3 | 1.2 | 0.8 | **6.3** | 0.4 | 5.9 | 7.8 | 20.8 | 28.2 | 30.6 | **79.6** |
| **Oxygen therapy at home** | 5.1 | 2.7 | 3.1 | **10.9** | 5.1 | 11.4 | 13.3 | 23.1 | 20.4 | 15.7 | 59.2 |
| **Severe cerebral paresis** | 7.5 | 2.7 | 3.5 | **13.7** | 6.3 | 16.1 | 11.0 | 22.4 | 20.8 | 9.8 | 53 |
| **Transplanted** | 7.8 | 2.4 | 3.1 | **13.3** | 5.9 | 14.9 | 11.4 | 19.2 | 20.8 | 14.5 | 54.5 |
| **High-risk interventions** | 7.1 | 1.6 | 2.7 | **11.4** | 2.4 | 8.6 | 10.6 | 16.9 | 25.1 | 25.1 | **67.1** |
| Central venous line | 10.6 | 1.6 | 4.3 | 16.5 | 5.9 | 13.7 | 12.9 | 19.6 | 23.1 | 8.2 | 509 |
| Age <1 | 9.8 | 3.9 | 2.7 | 16.4 | 5.1 | 12.9 | 11.0 | 16.9 | 22.0 | 15.7 | 54.6 |
| Previous involvement (P)ICU | 9.4 | 3.1 | 5.5 | 18 | 5.9 | 12.2 | 14.5 | 20.8 | 17.6 | 11.0 | 49.4 |
| Increscent medication per 24 hours | 11.0 | 3.1 | 5.9 | 20 | 8.2 | 18.8 | 14.1 | 18.8 | 14.1 | 5.9 | 38.8 |
| >3 specialisms involved in treatment | 11.8 | 4.7 | 6.3 | 22.8 | 8.2 | 20.0 | 14.1 | 18.4 | 11.4 | 5.1 | 34.9 |
| Communication problems | 11.4 | 5.5 | 5.1 | 22 | 7.1 | 17.6 | 12.2 | 22.0 | 15.3 | 3.9 | 41.2 |
| Stoma | 19.2 | 11.8 | 10.6 | 41.6 | 11.8 | 21.2 | 11.4 | 9.8 | 3.9 | 0.4 | 14.1 |
| Epilepsy | 11.4 | 5.1 | 6.7 | 23.2 | 0.5 | 19.6 | 12.5 | 20.4 | 11.8 | 5.1 | 37.3 |
| Anemia (Hb) (mmol/L) | 8.6 | 4.3 | 7.1 | 20 | 8.6 | 19.2 | 14.9 | 22.0 | 9.4 | 5.9 | 37.3 |
| Cognitive or genetic abnormalities | 8.6 | 5.1 | 8.2 | 21.9 | 6.3 | 18.4 | 14.9 | 17.6 | 12.5 | 8.2 | 38.3 |
| Blood cultures in previous 72 hours | 15.3 | 5.5 | 13.7 | 34.5 | 9.0 | 20.8 | 11.0 | 13.3 | 7.1 | 4.3 | 24.7 |

9-point Likert scale: 1 ‘not important’ to 9 ‘critically important’. Items in bold met criteria for inclusion.

**Appendix 7.** Measuring PEWS: who, how often, and when (n=256) (%)

| **In which children should PEWS be assessed?** |  |
| --- | --- |
| All hospitalized children ^1^ | 52.4 |
| Only children with unplanned admission^1^ | 18.4 |
| All children in a day admission^1^ | 2.4 |
| Only children hospitalized after a surgery with sedation^1^ | 14.8 |
| Only children hospitalized with monitoring/SaO_2_ monitoring^1^ | 25.6 |
| Depending on the first-line specialization^1^ | 4.8 |
| Don’t know | 0.4 |
| No preference | 0 |
| Other, …. | 31.6 |
| **How often should PEWS be measured?** |  |
| 1x/24h | 6.4 |
| 2x/24h | 6.4 |
| 3x/24h | 46 |
| No standard protocol | 10.8 |
| Other… | 30.8 |
| **When should PEWS be measured?** |  |
| At start of shift nurse^1^ | 45.6 |
| Not standardized^1^ | 29.6 |
| Other, …. | 17.6 |

^1^ Percentage ‘yes’ on statements (nine yes/no questions).
